# Pan-tumor survey of *ROS1* fusions detected by next-generation RNA and whole transcriptome sequencing

**DOI:** 10.1186/s12885-023-11457-2

**Published:** 2023-10-18

**Authors:** Misako Nagasaka, Shannon S. Zhang, Yasmine Baca, Joanne Xiu, Jorge Nieva, Ari Vanderwalde, Jeffrey J. Swensen, David Spetzler, Wolfgang Michael Korn, Luis E. Raez, Stephen V. Liu, Sai-Hong Ignatius Ou

**Affiliations:** 1 Department of Medicine, Division of Hematology and Oncology, University of California Irvine School of Medicine, 200 South Manchester Ave, Orange, CA 92868 USA; 2 Chao Family Comprehensive Cancer Center, Orange, CA USA; 3 Department of Internal Medicine, Division of Neurology, St. Marianna University School of Medicine, Kawasaki, Kanagawa Japan; 4 Caris Life Sciences, Phoenix, AZ USA; 5 USC Norris Comprehensive Cancer Center, University of Southern California Keck School of Medicine, Los Angeles, CA USA; 6 University of California San Francisco, San Francisco, CA USA; 7 Memorial Healthcare System/Florida Atlantic University, Pembroke Pines, FL USA; 8 Georgetown Lombardi Comprehensive Cancer Center, Georgetown University School of Medicine, Washington, DC USA

**Keywords:** c-ROS1, Receptor tyrosine kinase fusions, Non-small cell lung cancer, Breast cancer, Pan-tumor analysis

## Abstract

**Background:**

Two *ROS1* tyrosine kinase inhibitors have been approved for *ROS1* fusion positive (*ROS1*+) non-small cell lung cancer (NSCLC) tumors. We performed a pan-tumor analysis of the incidence of *ROS1* fusions to assess if more *ROS1*+ patients who could benefit from *ROS1* TKIs could be identified.

**Methods:**

A retrospective analysis of *ROS1* positive solid malignancies identified by targeted RNA sequencing and whole transcriptome sequencing of clinical tumor samples performed at Caris Life Science (Phoenix, AZ).

**Results:**

A total of 259 *ROS1*+ solid malignancies were identified from approximately 175,350 tumors that underwent next-generation sequencing (12% from targeted RNA sequencing [Archer]; 88% from whole transcriptome sequencing). *ROS1*+ NSCLC constituted 78.8% of the *ROS1*+ solid malignancies, follow by glioblastoma (GBM) (6.9%), and breast cancer (2.7%). The frequency of *ROS1* fusion was approximately 0.47% among NSCLC, 0.29% for GBM, 0.04% of breast cancer. The mean tumor mutation burden for all *ROS1*+ tumors was 4.8 mutations/megabase. The distribution of PD-L1 (22C3) expression among all *ROS1*+ malignancies were 0% (18.6%), 1%-49% (29.4%), and ≥ 50% (60.3%) [for NSCLC: 0% (17.8%); 1–49% (27.7%); ≥ 50% (53.9%).

The most common genetic co-alterations of *ROS1*+ NSCLC were *TP53* (29.1%), *SETD2* (7.3%), *ARIAD1A* (6.3%), and *U2AF1* (5.6%).

**Conclusions:**

*ROS1*+ NSCLC tumors constituted the majority of *ROS1*+ solid malignancies with four major fusion partners. Given that > 20% of *ROS1*+ solid tumors may benefit from *ROS1* TKIs treatment, comprehensive genomic profiling should be performed on all solid tumors.

**Supplementary Information:**

The online version contains supplementary material available at 10.1186/s12885-023-11457-2.

## Background

Receptor tyrosine kinase (RTK) fusion has been recognized as oncogenic structural gene rearrangements in solid malignancies [1]. Among the 58 human RTKs [2], there are US Food and Drug Administration (FDA) approved treatments in anaplastic lymphoma kinase (*ALK)*, c-ROS1 (*ROS1)*, rearranged in transformation (*RET)*, fibroblastic growth factor receptor (*FGFR2-3*), and neutrophin receptor tyrosine kinase (*NTRK1-3)* fusion positive tumors. With the exception of *NTRK* and *RET* fusions, all of the US FDA approvals are tumor-specific: *ALK* (non-small cell lung cancer [NSCLC]), *ROS1* (NSCLC), and *FGFR2-3* (urothelial, cholangiocarcinoma). Although these RTK fusions are found in all solid tumors albeit in a lower frequency, the main biology of the pathological process is univerval and not tumor histology-specific. Therefore, it is important to identify RTK fusions systematically beyond the specific tumor histologic types with approved treatments to expand the horizon of RTK fusion patients who may benefit from the expanded approval of treatments by raising awareness among clinicians, pharmaceutical companies and regulatory authorities to screen and enroll these patients in future clinical trials.

In this study, we performed a large-scale pan-tumor survey of *ROS1* fusions detected by next generation RNA sequencing to identify and characterize the molecular characteristics of *ROS1*+ solid tumors.

## Methods

### Patient cohort

A total of 259 *ROS1*+ tumors were identified in a retrospective assessment of a deidentified molecular profiling database surveyed for solid tumors that underwent fusion testing from a cohort including all cases submitted to a Clinical Laboratory Improvement Amendments (CLIA)–certified laboratory (Caris Life Sciences, Phoenix Arizona) for comprehensive genomic profiling. All unique cases that underwent successful fusion testing for targeted RNA sequencing were identified and included in this study.

This study was conducted in accordance with guidelines of the Declaration of Helsinki, Belmont report, and U.S. Common rule. In keeping with 45 CFR 46.101 (b) [4], this study was performed utilizing retrospective, deidentified clinical data. The need for written informed consent and ethical approval was waived by the University of California Irvine ethic committee due to the retrospective nature of the study. Table 1 shows the list of cancer type studied in this cohort.

**Table 1:** List of cancer types in studied cohort

Cancer Type	N
Non-small cell lung cancer (NSCLC)	204
High Grade Glioma	18
Breast Carcinoma	7
Pancreatic Adenocarcinoma	4
Ovarian	4
Cancer of Unknown Primary	3
Cholangiocarcinoma	3
Colorectal Adenocarcinoma	3
Gastric Adenocarcinoma	3
Sarcoma	3
Esophageal and Esophagogastric Junction Carcinoma	2
Bladder cancer—urothelial	1
Melanoma	1
Neuroendocrine tumors	1
Small Intestinal Malignancies	1
Thyroid Carcinoma	1
**Total**	**259**

### Fusion detection

Detailed methods on targeted RNA sequencing and whole transcriptome sequencing (WTS) have been previously described [3]. For tumors tested before February 2019, targeted RNA next generation-sequencing (NGS) was performed. For tumors tested after February of 2019, gene fusion detection was performed as part of whole transcriptome sequence (WTS) analysis on mRNA isolated from a formalin-fixed paraffin-embedded tumor sample using the Illumina NovaSeq platform (Illumina, Inc., San Diego, CA) and Agilent SureSelect Human All Exon V7 bait panel (Agilent Technologies, Santa Clara, CA). FFPE specimens underwent pathology review to diagnose percent tumor content and tumor size; a minimum of 10% of tumor content in the area for microdissection was required to enable enrichment and extraction of tumor-specific RNA. Qiagen RNA FFPE tissue extraction kit was used for extraction, and the RNA quality and quantity was determined using the Agilent TapeStation.

For tumors tested prior to February of 2019, anchored multiplex PCR was performed for targeted RNA NGS using the ArcherDx fusion assay (Archer FusionPlex Solid Tumor panel). The formalin-fixed paraffin-embedded tumor samples were microdissected to enrich the samples to ≥ 20% tumor nuclei, and mRNA was isolated and reverse transcribed into complementary DNA (cDNA). Unidirectional gene-specific primers were used to enrich for target regions, followed by NGS (Illumina MiSeq platform). Targets included 52 genes, and the full list can be found at http://archerdx.com/fusionplex-assays/solid-tumor. In the studied cohort, 55 samples were tested using ArcherDx fusion assay and 204 were testing using WTS.

### PD-L1 expression (TPS score)

NSCLC tumors tested after January of 2016 were stained with PD-L1 using primary PD-L1 antibody clone of 22c3 (Dako). Tumor Proportion Score (TPS) was measured, which is the percentage of viable tumor cells showing partial or complete membrane staining at any intensity. The tumor was considered positive if TPS ≥ 1% (high PD-L1 expression if TPS ≥ 50%).

### Tumor mutation burden (TMB)

TMB was measured (592 genes and 1.4 megabases [MB] sequenced per tumor) by counting all non-synonymous missense mutations found per tumor that had not been previously described as germline alterations according to dbSNP and 1 KG databases.

TMB was adjusted by dividing by a factor of 1.2 and a cutoff point of ≥ 10 mutations per MB was used based on the KEYNOTE-158 pembrolizumab trial [4], which showed that patients who had failed standard of care therapy and a TMB of ≥ 10 mt/MB across several tumor types had higher response rates than patients with a TMB of < 10 mt/MB. Caris Life Sciences is a participant in the Friends of Cancer Research TMB Harmonization Project [5].

### Survival and statistical analysis

Survival analysis was performed using real-world evidence from insurance claims data and calculated from time of tissue collection to last contact or time on treatment using Kaplan–Meier survival analysis. JMP statistical software was used to calculate mean/median tumor mutation burden (TMB), standard deviation (SD) and range as well as the mean junction read. Statistical significance was determined using chi-square and Wilcoxon rank sum test and adjusted for multiple comparisons.

## Results

### Incidence and distribution of *ROS1*+ solid tumors

A total of 259 *ROS1* fusions in solid tumors were detected by NGS RNA from 175,350 unique tumor samples composed of 16 different tumor types. Among these 175,350 tumor samples, 154,200 samples were profiled by WTS and 21,150 were profiled by NGS targeted RNA sequencing (Archer). Thus, the overall incidence of in-frame *ROS1*+ solid tumors was 0.15% (259/175350) by RNA NGS. The clinical characteristics of the three most common tumor type with *ROS1* fusions is summarized in Table 2.

**Table 2:** Patient and clinical characteristcis of *ROS1*+ solid tumors

	**All**	**NSCLC**	**Glioblastoma**	**Breast**	**Other**
**N (%)**	259	204 (79%)	18 (7%)	7 (3%)	30 (11%)
**Age median (range)**	63 (18–89)	65 (27–89)	63 (41–89)	60 (40–77)	52.5 (18–80)
**Male**	113 (44%)	86 (42%)	11 (61%)	0 (0%)	16 (53%)
**Female**	146 (56%)	118 (58%)	7 (39%)	7 (100%)	14 (47%)
**Sequencing methods**					
**Targeted RNA Archer (prior to 2019)**	55 (21%)	49 (24%)	4 (22%)	1 (14%)	1 (3%)
**WTS (post 2019)**	204 (79%)	155 (76%)	14 (78%)	6 (86%)	29 (96%)
**Mean junction read (SD)**	54.7	64 (107.8)	32.2 (55.1)	6.7 (5.2)	25.6 (25.8)
**Median** **TMB**	4	4	3	5	4

**Fig. 1:**
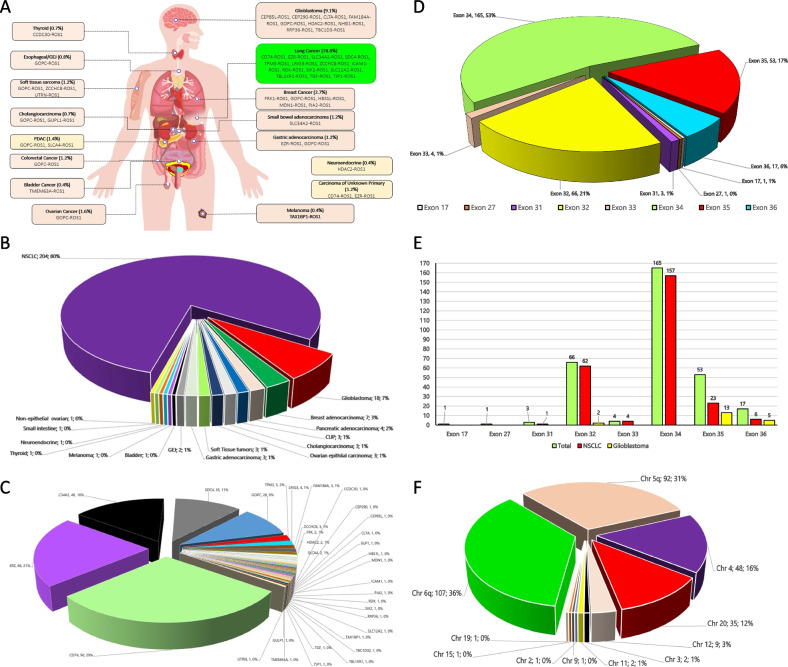
**A** Schematic diagram of the primary tumor site of *ROS1* fusions and the *ROS1* fusion variants identified in tumor site. **B** Pie-chart showing the distribution of the primary site of *ROS1* fusion positive tumors. **C** Pie-chart showing the distribution of fusion partners in all *ROS1*+ solid tumors. **D** Pie chart showing the frequency of fusion breakpoint by *ROS1* exons. **E** Comparison of *ROS1* exon fusion breakpoints overall and with NSCLC and glioblastoma. **F** Pie chart showing *ROS1*+ solid tumor chromosonal breakpoints

*ROS1*+ NSCLC tumors made up of 78.8% of the *ROS1*+ solid tumors, followed by *ROS1*+ GBM (6.9%), and *ROS1*+ breast cancer (2.7%) followed by 1–3 cases of *ROS1* fusions among the the rest of the 13 tumor types (Fig. 1A body figure, Fig. 1B pie chart). A total of 33 different fusion partners were identified among the *ROS1*+ solid tumors (Fig. 1C, Supplementary Table 1). The distribution of the fusions partners among the *ROS1*+ solid tumors are shown in Fig. 1C. The distribution of the exon breakpoints among the *ROS1*+ solid tumors are shown in Fig. 1D and A comparison of overall with NSCLC and GBM are showin in Fig. 1E. The chromosomal breakpoints of *ROS1*+ solid tumors are shown in Fig. 1F.

### *ROS1*+ NSCLC tumors

**Fig. 2:**
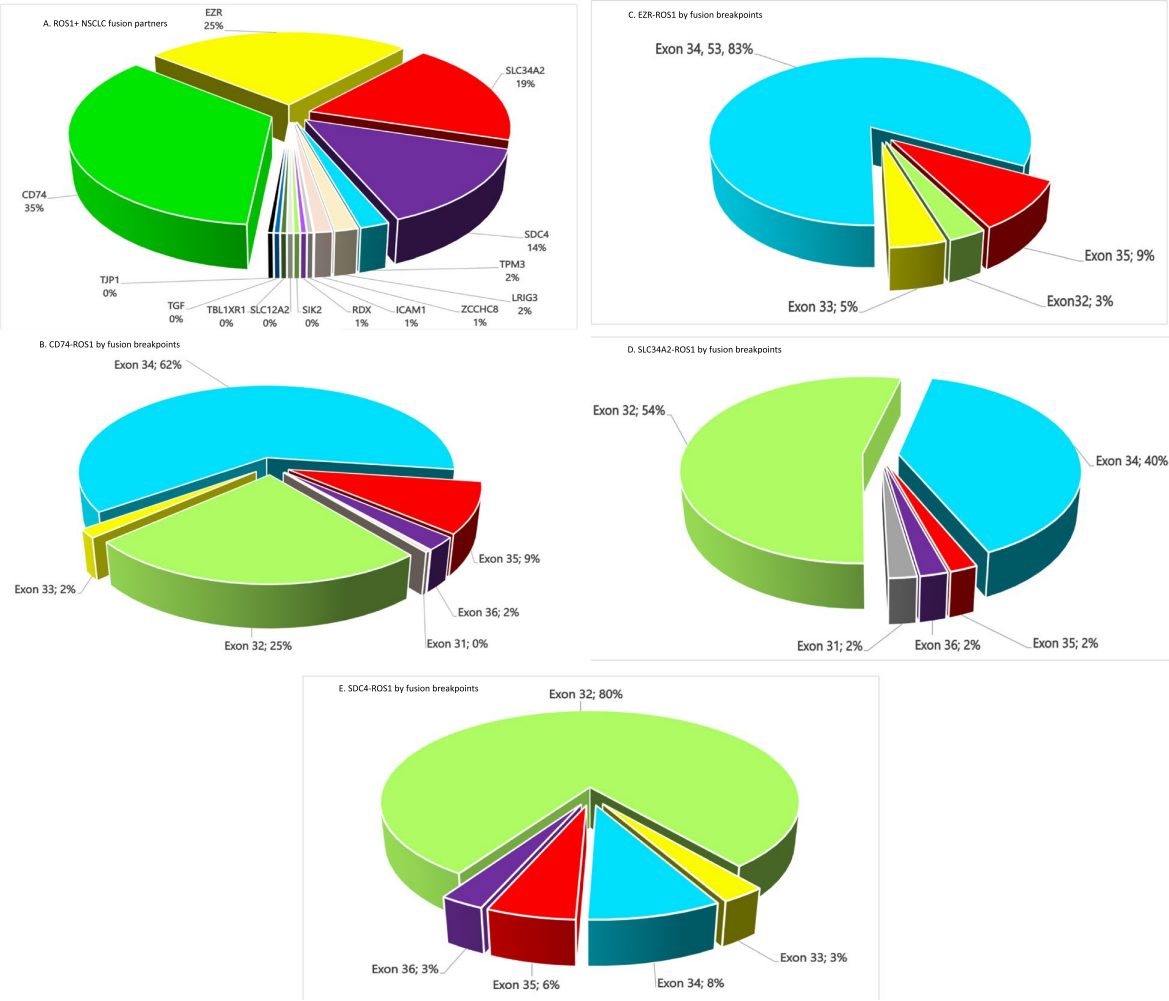
**A** Pie chart showing the distribution of fusions partners in *ROS1*+ NSCLC. **B** Pie chart showing the frequency of fusion breakpoint by *ROS1* exons in *CD74-ROS1*+ *.* **C** Pie chart showing the frequency of fusion breakpoint by *ROS1* exons in *EZR-ROS1*+ *.* **D** Pie chart showing the frequency of fusion breakpoint by *ROS1* exons in *SLC34A2-ROS1*+ *.* **E** Pie chart showing the frequency of fusion breakpoint by *ROS1* exons in *SDC4-ROS1*+

Among the 259 *ROS1*+ tumors, 204 (78.8%) were *ROS1*+ NSCLC (Fig. 1B). The overall incidence of *ROS1*+ NSCLC was approximately 0.47% (204/43404) in the database. There was no difference in the detection rates of *ROS1*+ NSCLC by ArcherDx fusion assay (0.52%, 49/9393) and WTS (0.55%, = 155/28173). Four fusions partners essentially made up the bulk of the *ROS1*+ NSCLC with *CD74-ROS1* (34.8%) being the most common fusion variant followed by *EZR-ROS1* (25.3%), *SLC34A2-ROS1* (18.6%), and *SDC4-ROS1* (13.8%) (Fig. 2A).

The vast majority of exon breakpoints at *ROS1* spanned 4 exons (exons 32, 34, 35, 36) with exon 34 (62.1%) being the most common breakpoint (Fig. 1D). There seemed to be a correlation between exon breakpoints and the fusion partners. *CD74-ROS1* and *EZR-ROS1* fusions were more commonly generated from breakpoint at *ROS1* exon 34 (Fig. 2B and C) while *SLC34A2-ROS1* and *SDC4-ROS1* fusions were generated more commonly with ROS1 exon 32 (Fig. 2D and E). Interestingly, the much rarer *LRIG3-ROS1* and *TPM3-ROS1* fusion variants were generated from breakpoint at *ROS1* exon 35 (Supplementary Table 2). Importantly, the *ROS1* fusion breakpoint at exon 35 or 36 both encode the transmembrane region of the *ROS1* protein. The transmembrane domain of *ROS1* protein spans amino acids 1862–1883 and the cytoplasmic domain which contain the kinase domain from 1884–2347. The kinase domain is between amino acids 1945–2222 with the ATP binding site within 1951–1980. (https://www.ebi.ac.uk/interpro/protein/reviewed/P08922/) accessed October 25, 2020).

Molecularly, the exon fusion breakpoints in exons 32, 34–36 with fusion at exon 34 was the most common especially among *ROS1*+ NSCLC tumors while exon 35 were found as fusion breakpoints for *ROS1*+ non-NSCLC tumors (Fig. 1E). Among the junctional reads of the major *ROS1*+ tumors, the highest was among NSCLC, followed by GBM. Of note, *ROS1*+ breast adenocarcinoma, though with limited number of samples, had a tenfold lower junction reads than those of *ROS1*+ NSCLC (Supplementary Table 3).

In terms of biomarkers for potential efficacy for immune checkpoint inhibitors. The median tumor mutation burden (TMB) for *ROS1*+ NSCLC was 4, the mean was 4.8 (SD 2.8, range 0–15) and only 4.6% of *ROS1*+ NSCLC tumors had TMB >  = 10. The percentage of tumor samples with PD-L1 expression >  = 50% was 54.5% and >  = 1% was 81.2% (Supplementary Fig. 1).

### *ROS1*+ GBM

**Fig. 3:**
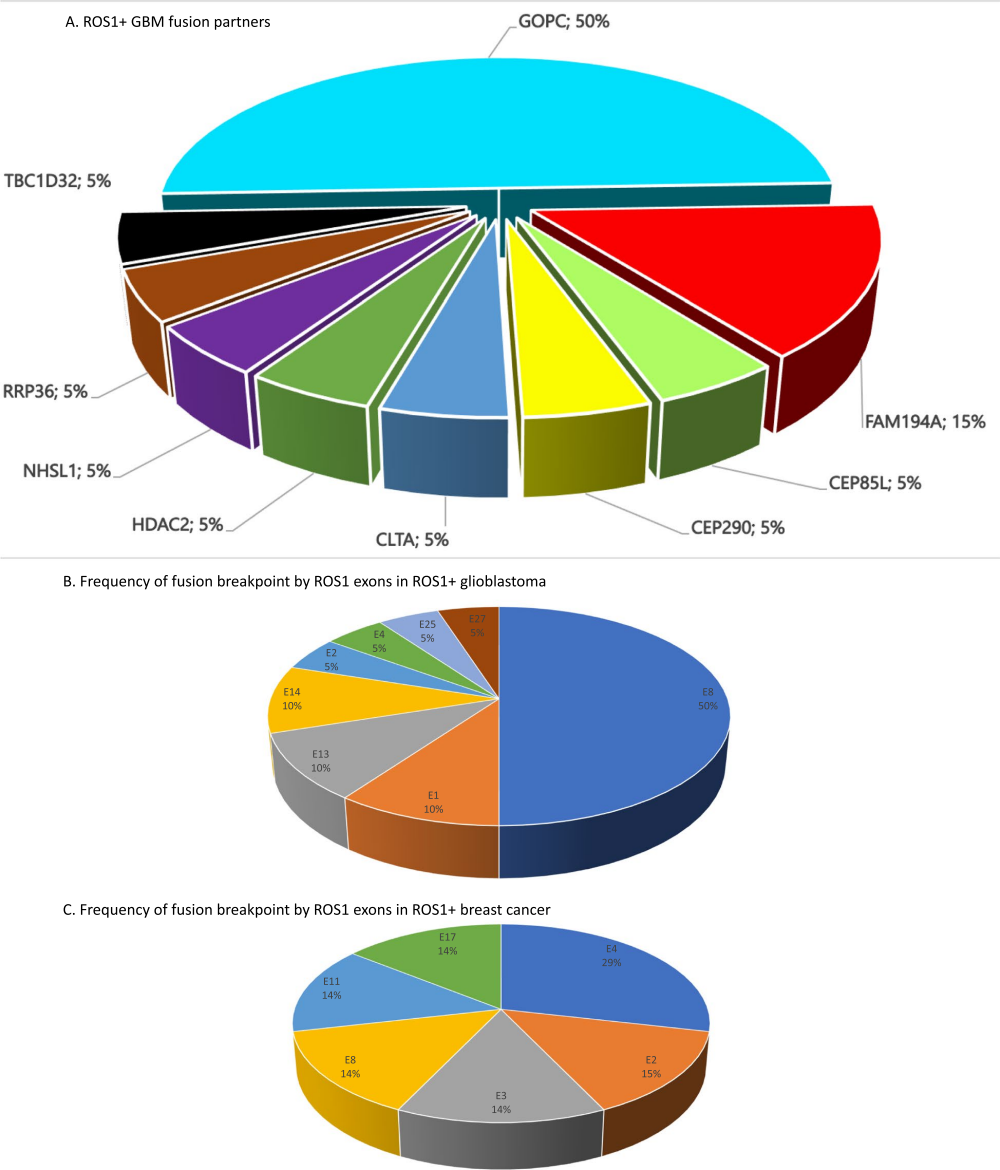
**A** Pie chart showing the frequency of fusion partners in *ROS1*+ glioblastoma multiforme. **B** Pie chart showing the frequency of fusion breakpoint by *ROS1* exons in *ROS1*+ glioblastoma multiforme. **C** Pie chart showing the frequency of fusion breakpoint by *ROS1* exons in *ROS1*+ breast cancers

Glioblastoma was the second most common *ROS1*+ fusion tumors with an incidence of 6.9% (18/259) in this pan-tumor survey. The incidence of *ROS1*+ GBM was approximately 0.29% (18/6206). GOPC was the most common fusion partner (50%) (Fig. 3A). Figure 3B shows the frequency of fusion breakpoint by *ROS1* exons in *ROS1*+ GBM.

All of the GBM samples had TMB < 10 mt/MB with a median TMB of 3 (Table 2). The majority of GBM had no PD-L1 expression (66.7%) and no samples had a PD-L1 expression >  = 50%.

### *ROS1*+ breast cancer tumors

*ROS1*+ breast cancer is the third most common *ROS1* fusion constituting 2.7% of the *ROS1*+ solid tumors (Fig. 1B). The incidene of *ROS1*+ breast cancer in the database was approximately 0.04% (7/17500). The hormonal status of the five *ROS1*+ breast cancer cases are listed in Table 3. One of the *GOPC-ROS1* had an unique exon breakpoint at exon 17 of *ROS1* (Fig. 3C).

**Table 3:** Hormonal receptor status of the 7 *ROS1*+ breast cancers

Number	***ROS1*** fusion	Hormonal status
1	*GPOC-ROS1* (G8, R35)	ER + , PR + , HER2-
2	*GOPC-ROS1* (G3, R36)	ER-, PR-, HER2- (triple negative)
3	*FRK-ROS1* (F2, R34)	Unknown (not done?)
4	*FRK-ROS1* (F4, R32)	ER + , PR + , HER2 +
5	*MDN1-ROS1* (M17, R17)	ER-, PR-, HER2- (triple negative)
6	*PJA2-ROS1* (P4, R36)	ER-, PR-, HER2- (triple negative)
7	*HBS1L-ROS1* (H11, R27)	ER-, PR-, HER2 +

### Mean allele (fusion) frequency

The mean number of junctional read among all tumor types was 54.7 with NSCLC at 64.0 (SD 107.77), glioblastoma at 32.2 (SD55.12) and breast cancer at 6.7 (SD 5.20) (Table 2).

### Co-occurrence with other genetic aberrations

*ROS1*+ NSCLC were mutually exclusive with known actionable oncogenic alterations in *EGFR, KRAS, ALK, RET, NTRK* or *NRG*. *TP53* mutations (29.1%) were the most common co-mutations followed by *SETD2* mutations (7.3%), *ARIAD1A* mutations (6.3%) and *U2AF1* (5.6%). The complete list of genetic co-alterations are listed in (Supplementary Table 4).

### PD-L1 expression

The PD-L1 expression status were determined in 191 out of the 204 *ROS1*+ NSCLC samples. When the 191 *ROS1*+ NSCLC samples with known PD-L1 status were analyzed, the distribution by PD-L1 expression were: 0% (*n* = 34), 1–49% (53), and > 50% (104) (Supplementary Table 5). When the 204 *ROS1*+ tumor samples with known PD-L1 status were analyzed, the distribution by PD-L1 expression were: 0% (*n* = 38), 1–49% (*n* = 60), and > 50% (106) (Supplementary Table 6) and were heavily impacted by NSCLC data. 18 patients with *ROS1*+ glioblastoma had PD-L1 testing by SP142 and 33.3% (*n* = 6/18) were positive while 4 patients with *ROS1*+ breast cancer had PD-L1 testing by SP142 and 25% (*n* = 1/4) were positive (data not shown).

### Tumor mutation burden

The median TMB was 4 mutations/MB for all ROS1+ tumors (*n* = 259) while it was 4 in NSCLC, 3 in GBM and 5 in breast cancer patients with *ROS1* fusions (Table 2).

### Real world survival data

**Fig. 4:**
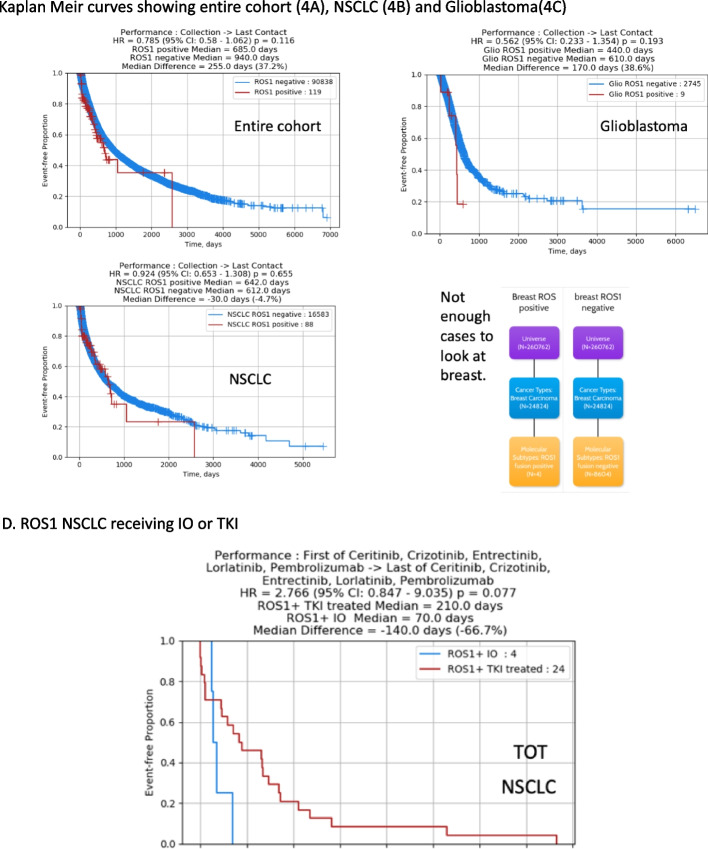
**A** Kaplan Meir curves showing *ROS1* positive vs *ROS1* negative in the entire cohort. **B** Kaplan Meir curves showing *ROS1* positive vs *ROS1* negative in the NSCLC cohort. **C** Kaplan Meir curves showing *ROS1* positive vs *ROS1* negative in the glioblastoma cohort. **D** Kaplan Meir curves showing time on treatment of *ROS1*+ NSCLC patients receiving *ROS1* TKIs versus immunotherapy

Kaplan-Meir curves showing time from sample collection to last contact is shown in Fig. 4A-C. There was no significant statistical difference seen in the entire cohort (Fig. 4A), NSCLC (Fig. 4B) and GBM patients (Fig. 4C) stratified by *ROS1* positivity. There were only 4 *ROS1*+ breast cancer patients who had survival outcome information and therefore insufficient to compare with the *ROS1-* patients. Despite the limited number of *ROS1*+ glioblastoma with OS survival information(*N* = 9), *ROS1*+ GBM patients had overall approximately 6 months (170 days) shorter OS while there was no major the differences between *ROS1*+ NSCLC versus non-*ROS1*+ NSCLC.

The Kaplan-Meir curve showing time on treatment showed a non-statistical significant trend of worse outcomes with immunotherapy use versus TKI use with a median number of treatment days of 210.0 days for *ROS1* positive NSCLC patients receiving TKIs (ceritinib, crizotinib, entrectinib, lorlatinib) versus median number of 70.0 days on treatment for *ROS1* positive NSCLC patients receiving immunotherapy with pembrolizumab (HR = 2.766, 95% CI 0.847–9.305, *p* = 0.077) although the sample size was limited (Fig. 4D).

## Discussion

In this first large scale survey of *ROS1* fusions identified by RNA NGS where only in-frame messenger RNA (mRNA) transcripts were reported, we identified 259 *ROS1*+ tumor samples by RNA NGS of tumor samples spanning 16 tumor types. Many inter/intragenic rearrangements have been reported in the literature using pure DNA NGS, but whether an in-frame mRNA were eventually transcribed (and the exact *ROS1* fusion variant) remained to be determined especially if clinical response was not reported [6, 7].

From the example of *NTRK* fusions, it is generally accepted that RTK fusions are likely an universal actionable driver among the vast majority if not all tumor types harboring that RTK fusion [1]. While *ROS1*+ NSCLC tumors were the dominant tumor type at 78.8% and has US FDA approved treatment of two *ROS1* TKIs [8, 9], it implies that potentially more than 20% of the *ROS1*+ solid tumor may benefit from currently approved *ROS1* TKIs.

Importantly, *ROS1*+ GBM constituted the second largest *ROS1*+ tumors at 6.9%. In fact *ROS1* fusion was first identified in glioblastoma multiforme in 1987 [10]. Thus, it not surprising that *ROS1*+ GBM constituted the second most common *ROS1*+ solid tumors. Although limited by the numbers of *ROS1*+ glioblastoma identified and thus statistically not significant, the presence of *ROS1* fusions in glioblastoma seems to indicate a poor prognosis. Entrectinib, a first-generation *ROS1* TKI, has CNS activity and second generation *ROS1* TKIs in clinical development such as repotrectinib, taletrectinib and NVL-520, also has either demonstrated CNS activity clinically or in pre-clinical models and thus could be considered as a potential treatment option for *ROS1*+ GBM patients [9, 11–13].

*ROS1*+ breast adenocarcinoma was the third most common *ROS1*+ solid tumors but only at 3%. Other *ROS1* fusion positive tumors that have been previously reported in the literature such as *ROS1*+ melanoma [14] and ROS1+ soft tissue tumor (inflammatory myofibroblastic tumor [IMT]; [15] were also identified in the database.

Molecularly, the exon fusion breakpoints in exons 32, 34–36 with fusion at exon 34 was the most common especially among *ROS1*+ NSCLC while exon 35 were found as fusion breakpoints for *ROS1*+ non-NSCLC tumors. Among the junctional reads of the major *ROS1*+ tumors, the highest was among NSCLC, followed by GBM. Of note, *ROS1*+ breast adenocarcinoma, although with limited number of samples, had a tenfold lower junction reads than those of *ROS1*+ NSCLC. Additionally, two *ROS1*+ breast adenocarcinoma had fusion breakpoints at exon 17 and exon 27, respectively.

In our study, *ROS1* fusions were also detected at similar frequency as previously reported *ROS1*+ NSCLC tumors of approximately 2% [16] in other major tumors such as breast and pancreatic cancers. This is consistent with prior reports of identification of *ROS1* beyond NSCLC [17–24]. While the second most common tumor type glioblastoma is considered rare, the third most common tumor type in our study was breast cancer, which is one of the most common types of tumor. A previous Chinese study of 1440 breast cancer patients described a total of 30 RTK events including 3 with *ROS1* [18]. Although the hormonal status of these patients were not described in the paper, a prior case report on inflammatory breast cancer harboring *CD74-ROS1* was triple negative [17] and so were 3 out of 7 cases of *ROS1*+ breast cancer in our study. Triple negative breast cancer patients are known to have less treatment options. Thus, it is important to profile tumors beyond NSCLC for *ROS1* fusions given there are now two approved tyrosine kinase inhibitors for the treatment of *ROS1*+ NSCLC.

By far, the most common *ROS1*+ tumor type was NSCLC. We identified 8 of the 24 *ROS1*+ NSCLC fusion partners reported in the literature [25]. However, 4 (CD74, EZR, SLC34A2 and SDC4) of the fusion partners made up the vast majority of the *ROS1*+ NSCLC fusion partners. Neel et al. have demonstrated that different fusion partners affect the subcellular localization of the *ROS1* fusions [26] while Li et al. has described that *ROS1*+ NSCLC patients with *CD74-ROS1* fusion partners are more likely to present with brain metastases and showed a trend toward improved survival in the non-*CD74-ROS1* group when they were treated with crizotinib [27], suggesting the possibility that fusion partners may have differential responses to therapy. As in the case with *ALK*-rearranged NSCLC, concurrent mutations such as *TP53* may also play a role on differential responses to targeted therapy [28] and further exploration in the space of fusion partners and concurrent mutations in *ROS1*+ NSCLC is eagerly awaited.

Also notable in this brief report is the fact that to our knowledge, this is the first large scale survey of PD-L1 expression among *ROS1*+ NSCLC. PD-L1 expression was detected in 81.2% of the *ROS1*+ NSCLC samples where the PD-L1 expression was known. The majority of the PD-L1 positive *ROS1*+ NSCLC (> = 1%) were high expressors (54.5%, 104/191). In these patients, clinicians may be tempted to use single agent pembrolizumab as the first-line treatment of *ROS1*+ NSCLC given the overall survival benefit of Keynote-024 results for PD-L1 expression (> = 50%) and the FDA expanded approval of pembrolizumab approval of pembrolizumab for PD-L1 >  = 1% based on the Keynote-042 results as only *EGFR* + and *ALK* + NSCLC were excluded and *ROS1*+ NSCLC were not excluded from these studies [29, 30].

However, single agent immune checkpoint inhibitor appears to have limited activity in actionable driver mutation positive NSCLC. It is generally recognized that single agent immunotherapy is not effective in *EGFR* mutated NSCLC [31, 32]. Although evidence in *ROS1* fusion positive NSCLC is limited, a global registry has shown limited ORR of immune checkpoint inhibitors in NSCLC harboring oncogenic alterations with reported ORR of *ROS1* fusion + patients being 17% (*n* = 7) and 12% (*n* = 125) for *EGFR* mutated patients [33]. While better than the ORR of 12% in *EGFR* mutated patients, immunotherapy as a single agent may not be as effective as other options in *ROS1* fusion + NSCLC. Although statistically non-significant and limited analysis due to small sample size, our study also showed that the time on treatment was less with immunotherapy versus *ROS1* targeted TKIs in *ROS1*+ NSCLC. Further prospective data on efficacy as well as safety are warrented.

Overall the incidence of *ROS1*+ NSCLC detected in this database was lower than the generally accepted approximately 2% incidence in the literature [16]. Targted RNA NGS and WTS are the most vigorous platforms in detecting RTK fusions where the transcribed RNA are detected and the reading frame is checked to ensure it is "in frame". In this study, there was no difference in the detection rates of *ROS1*+ by ArcherDx fusion assay (0.52%, 55/9393 and WTS (0.55%, 155/28173) in the NSCLC cohort which constituted the majority of the *ROS1* fusions.

One of the limitations of this study is the fact that there may be selection bias in those who were offered molecular testing. This is likely due to selection biases as *ROS1* fusions may be detected first by FISH and DNA NGS and RNA NGS are likely being employed when the tumor are “pan-negative”. Additionally, there may have been further selection bias based on the baseline characteristics of patients such as smoking status, age, gender, and histology (i.e. in NSCLC, adenocarcinoma may likely be offered NGS more frequently than other histologies). Another limitation of this study is the lack of detailed clinical information regarding the timing of when the molecular analysis was performed (i.e. stage, pre vs post treatment evaluation). Outcomes were inferred based on time from tissue collection to date of last contact or time on treatment. In reality, NGS is performed at varying time points during the course of the disease and treatments.

## Conclusions

Despite limitations, we were able to determine the characteristics of *ROS1* fusions in a tumor agnostic manner. *ROS1* fusions were identified in multiple tumor types. Only 78.8% with NSCLC have approved *ROS1* targeted therapy. Further studies to investigate the role of tyrosine kinase inhibitors in the approximately one fifth of solid tumors with *ROS1* fusions would be warranted. Finally, a more detailed examination of the clinical effects of other co-existing mutations along with underlying biological and molecular mechanism including high PDL1 score and having concurrent alterations such as *TP53* to account for the differences in survival outcomes of various *ROS1* fusions is eagerly awaited.

### Supplementary Information

**Additional file 1:** **Supplementary Figure 1.** TPS score and TMB in ROS1+ NSCLC cohort.

**Additional file 2:** **Supplementary Table 1.** ROS1 fusions among solid tumors. **Supplementary Table 2.** Fusion partners and ROS1 exons. **Supplementary Table 3.** Mean Junction read among tumor types. **Supplementary Table 4.** Co-occurring alterations in ROS1+ NSCLC. **Supplementary Table 5.** Distribution of PDL1 TPS score among ROS1+ NSCLC. **Supplementary Table 6.** Distribution of PDL1 TPS score among ROS1+ tumors.

## Data Availability

All data generated or analysed during this study are included in this manuscript and its supplementary files. The datasets generated during and/or analyzed during the current study are available from the corresponding author on reasonable request. The deidentified sequencing data are owned by Caris Life Sciences, and cannot be publicly shared due to the data usage agreement signed by Dr. Sai-Hong Ignatius Ou. Qualified researchers can apply for access to these summarized data by contacting Joanne Xiu, PhD (jxiu@carisls.com) and signing a data usage agreement. The authors will honor legitimate requests for data sharing to qualified researchers, upon request, as necessary for conducting a methodologically sound research.

## Abbreviations

ALK: Anaplastic lymphoma kinase
ATP: Adenosine triphosphate
CI: Confidence interval
CLIA: Clinical laboratory improvement amendments
CNS: Central nervous system
DNA: Deoxyribonucleic acid
FDA: Food and Drug Administration
FFPE: Formalin-fixed paraffin embedded
FGFR: Fibroblastic growth factor receptor
FISH: Fluorescence in situ hybridization
EGFR: Epidermal growth factor receptor
GBM: Glioblastoma multiforme
IMT: Imflammatory myofibroblastic tumor
IRB: Institutional review board
MB: Megabase
NGS: Next generation sequencing
NSCLC: Non-small cell lung cancer
NTRK: Neutrophin receptor tyrosine kinase
PCR: Polymerase chain reaction
PD-L1: Programmed cell death ligand 1
RET: Rearranged in transformation
RNA: Ribonucleic acid
RTK: Receptor tyrosine kinase
ROS1: C-ROS1
TKI: Tyrosine kinase inhibitor
TMB: Tumor mutation burden
TPS: Tumor proportion score
WTS: Whole transcriptome sequencing
