# Supplementary material for: Pan-tumor survey of ROS1 fusions detected by next-generation RNA and whole transcriptome sequencing
Source: BMC Cancer. 2023 Oct 18;23:1000. doi: 10.1186/s12885-023-11457-2 (PMC10585918; doi:10.1186/s12885-023-11457-2)
Supplement: Supplementary file 1 — Additional file 1: Supplementary Figure 1. TPS score and TMB in ROS1+ NSCLC cohort. [file 12885_2023_11457_MOESM1_ESM.pdf]

Supplementary Figure 1. Supplementary Figure 1. TPS score and TMB in ROS1+ NSCLC cohort

TPS score in the ROS1+ NSCLC cohort

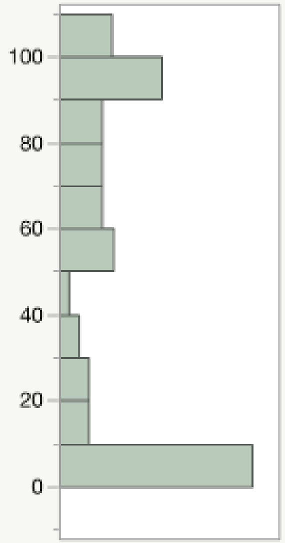

Test	Positive	Negative	Total	%
IHC-PD-L1 (22c3)	155	36	191	81.2%

Quantiles

100.0%	maximum	100
99.5%		100
97.5%		100
90.0%		95
75.0%	quartile	90
50.0%	median	50
25.0%	quartile	2
10.0%		0
2.5%		0
0.5%		0
0.0%	minimum	0

Summary Statistics

Mean	46.036649
Std Dev	38.354904
Std Err Mean	2.7752634
Upper 95% Mean	51.510935
Lower 95% Mean	40.562364
N	191

TMB in the ROS1+ NSCLC cohort

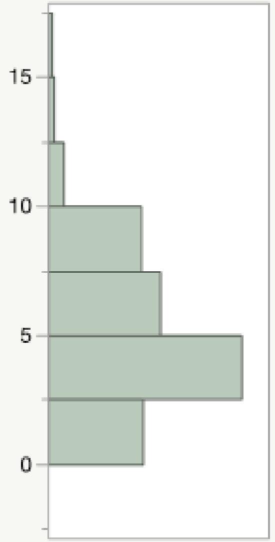

Test	Positive	Negative	Total	%
NGS -TMB	8	166	174	4.6%

Quantiles

100.0%	maximum	15
99.5%		15
97.5%		12
90.0%		8
75.0%	quartile	7
50.0%	median	4
25.0%	quartile	3
10.0%		2
2.5%		1
0.5%		0
0.0%	minimum	0

Summary Statistics

Mean	4.8563218
Std Dev	2.8481898
Std Err Mean	0.2159207
Upper 95% Mean	5.2825
Lower 95% Mean	4.4301437
N	174
