# Supplementary material for: Pan-tumor survey of ROS1 fusions detected by next-generation RNA and whole transcriptome sequencing
Source: BMC Cancer. 2023 Oct 18;23:1000. doi: 10.1186/s12885-023-11457-2 (PMC10585918; doi:10.1186/s12885-023-11457-2)
Supplement: Supplementary file 2 — Additional file 2: Supplementary Table 1. ROS1 fusions among solid tumors. Supplementary Table 2. Fusion partners and ROS1 exons. Supplementary Table 3. Mean Junction read among tumor types. Supplementary Table 4. Co-occurring alterations in ROS1+ NSCLC. Supplementary Table 5. Distribution of PDL1 TPS score among ROS1+ NSCLC. Supplementary Table 6. Distribution of PDL1 TPS score among ROS1+ tumors. [file 12885_2023_11457_MOESM2_ESM.docx]

**Supplementary Table 1.** *ROS1* fusions among solid tumors

**Supplementary Table 2.** Fusion partners and *ROS1* exons

**Supplementary Table 3.** Mean Junction read among tumor types

**Supplementary Table 4.** Co-occurring alterations in *ROS1+* NSCLC

**Supplementary Table 5.** Distribution of PDL1 TPS score among *ROS1+* NSCLC

**Supplementary Table 6.** Distribution of PDL1 TPS score among *ROS1+* tumors

Supplementary Table 1. ROS1 fusions among solid tumors


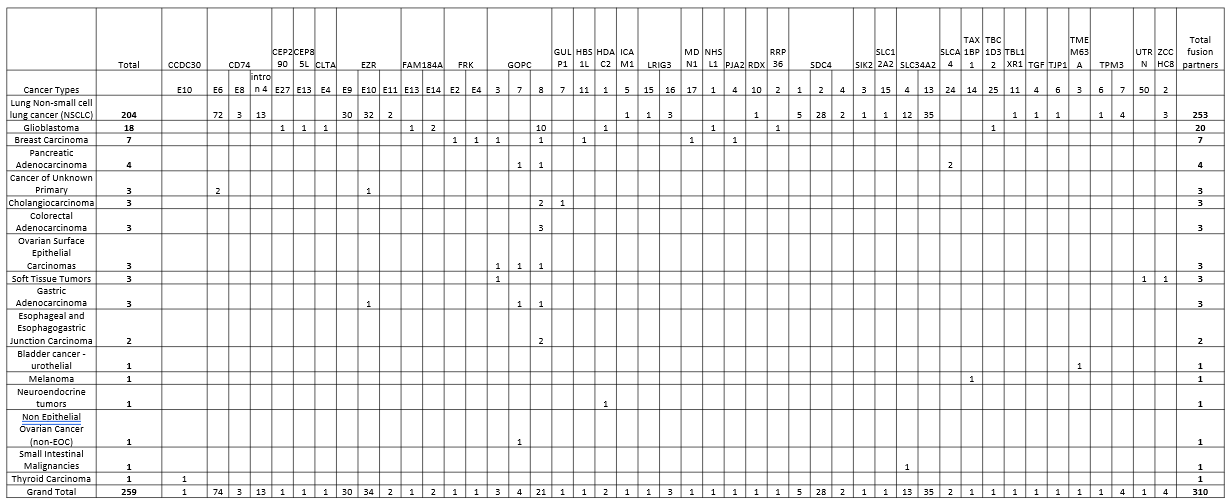


Supplementary Table 2. Fusion partners and ROS1 exons

|  |  | ROS1 exon | | | | | |  |
| --- | --- | --- | --- | --- | --- | --- | --- | --- |
|  |  | E31 | E32 | E33 | E34 | E35 | E36 | Total |
| CD74 | E6 |  | 2 |  | 65 | 5 |  | 72 |
|  | E8 |  |  |  | 3 |  |  | 3 |
|  | intron 4 |  |  |  | 13 |  |  | 13 |
| EZR | E9 |  |  | 3 | 23 | 4 |  | 30 |
|  | E10 |  | 2 |  | 28 | 2 |  | 32 |
|  | E11 |  |  |  | 2 |  |  | 2 |
| ICAM1 | E5 |  | 1 |  |  |  |  | 1 |
| LRIG3 | E15 |  |  |  |  | 1 |  | 1 |
|  | E16 |  |  |  |  | 3 |  | 3 |
| RDX | E10 |  |  |  | 1 |  |  | 1 |
| SDC4 | E1 |  | 2 |  | 2 |  | 1 | 5 |
|  | E2 |  | 24 | 1 | 1 | 2 |  | 28 |
|  | E4 |  | 2 |  |  |  |  | 2 |
| SIK2 | E3 |  | 1 |  |  |  |  | 1 |
| SLC12A2 | E15 |  |  |  |  |  | 1 | 1 |
| SLC34A2 | E4 | 1 | 7 |  | 3 | 1 |  | 12 |
|  | E13 |  | 19 |  | 16 |  |  | 35 |
| TBL1XR1 | E11 |  |  |  |  | 1 |  | 1 |
| TGF | E4 |  |  |  |  | 1 |  | 1 |
| TJP1 | E6 |  | 1 |  |  |  |  | 1 |
| TPM3 | E6 |  |  |  |  | 1 |  | 1 |
|  | E7 |  | 1 |  |  | 2 | 1 | 4 |
| ZCCHC8 | E2 |  |  |  |  |  | 3 | 3 |
| Total |  | 1 | 62 | 4 | 157 | 23 | 6 | 253 |

Supplementary Table 3 Mean Junction read among tumor types

| All tumor types | Mean Junction read | N | SD |
| --- | --- | --- | --- |
| Neuroendocrine tumors | 76.0 | 1 | NA |
| Lung Non-small cell lung cancer (NSCLC) | 64.0 | 155 | 107.77 |
| Non Epithelial Ovarian Cancer (non-EOC) | 61.0 | 1 | NA |
| Cancer of Unknown Primary | 50.3 | 3 | 39.70 |
| Colorectal Adenocarcinoma | 37.3 | 3 | 8.08 |
| Glioblastoma | 32.2 | 14 | 55.12 |
| Pancreatic Adenocarcinoma | 25.0 | 4 | 34.75 |
| Gastric Adenocarcinoma | 23.7 | 3 | 30.60 |
| Ovarian Surface Epithelial Carcinomas | 21.7 | 3 | 15.37 |
| Esophageal and Esophagogastric Junction Carcinoma | 15.0 | 2 | 16.97 |
| Thyroid Carcinoma | 12.0 | 1 | NA |
| Soft Tissue Tumors | 9.7 | 3 | 8.14 |
| Cholangiocarcinoma | 8.3 | 3 | 5.51 |
| Breast Carcinoma | 6.7 | 6 | 5.20 |
| Bladder cancer - urothelial | 6.0 | 1 | NA |
| Melanoma | 5.0 | 1 | NA |
| Small Intestinal Malignancies | NA | 1 | NA |
| Total mean | 54.7 | 204 |  |

Supplementary Table 4 Co-occurring alterations in ROS1+ NSCLC

| Mutations | Positive | Negative | Total | % |
| --- | --- | --- | --- | --- |
| NGS -TP53 | 51 | 124 | 175 | 29.1% |
| NGS -SETD2 | 13 | 164 | 177 | 7.3% |
| NGS -LOH | 7 | 89 | 96 | 7.3% |
| NGS -ARID1A | 8 | 119 | 127 | 6.3% |
| NGS -U2AF1 | 10 | 170 | 180 | 5.6% |
| NGS -TERT | 5 | 97 | 102 | 4.9% |
| NGS -CDKN2A | 6 | 172 | 178 | 3.4% |
| NGS -HRR | 3 | 87 | 90 | 3.3% |
| NGS -SMAD4 | 6 | 174 | 180 | 3.3% |
| NGS -KMT2C | 5 | 158 | 163 | 3.1% |
| CNA-MYC | 5 | 171 | 176 | 2.8% |
| NGS -BAP1 | 5 | 173 | 178 | 2.8% |
| NGS -ATM | 5 | 175 | 180 | 2.8% |
| NGS -CREBBP | 5 | 175 | 180 | 2.8% |
| NGS -KDM5C | 4 | 144 | 148 | 2.7% |
| NGS -RB1 | 3 | 119 | 122 | 2.5% |
| NGS -EP300 | 4 | 175 | 179 | 2.2% |
| NGS -MET | 4 | 176 | 180 | 2.2% |
| NGS -SMARCA4 | 4 | 176 | 180 | 2.2% |
| NGS -FANCC | 3 | 174 | 177 | 1.7% |
| NGS -TET2 | 3 | 174 | 177 | 1.7% |
| NGS -PIK3CA | 3 | 176 | 179 | 1.7% |
| NGS -APC | 3 | 177 | 180 | 1.7% |
| NGS -CTNNB1 | 3 | 177 | 180 | 1.7% |
| NGS -NF2 | 3 | 177 | 180 | 1.7% |
| NGS -ASXL1 | 2 | 123 | 125 | 1.6% |
| NGS -SOS1 | 1 | 62 | 63 | 1.6% |
| NGS -RBM10 | 1 | 65 | 66 | 1.5% |
| CNA-ADGRA2 | 1 | 77 | 78 | 1.3% |
| CNA-CBFA2T3 | 1 | 77 | 78 | 1.3% |
| CNA-CYP2D6 | 1 | 77 | 78 | 1.3% |
| CNA-AXIN1 | 1 | 78 | 79 | 1.3% |
| CNA-HNRNPA2B1 | 1 | 78 | 79 | 1.3% |
| CNA-LGR5 | 1 | 78 | 79 | 1.3% |
| CNA-TRAF7 | 1 | 78 | 79 | 1.3% |
| CNA-ZNF703 | 1 | 78 | 79 | 1.3% |
| NGS -PRKDC | 2 | 166 | 168 | 1.2% |
| NGS -RAD50 | 2 | 166 | 168 | 1.2% |
| NGS -JAK2 | 2 | 168 | 170 | 1.2% |
| NGS -BCOR | 2 | 169 | 171 | 1.2% |
| NGS -CHEK2 | 2 | 172 | 174 | 1.1% |
| CNA-MDM2 | 2 | 173 | 175 | 1.1% |
| NGS -ARID2 | 2 | 173 | 175 | 1.1% |
| NGS -KMT2D | 2 | 173 | 175 | 1.1% |
| NGS -FH | 2 | 178 | 180 | 1.1% |
| NGS -MUTYH | 2 | 178 | 180 | 1.1% |
| NGS -PPP2R1A | 2 | 178 | 180 | 1.1% |
| NGS -FANCI | 1 | 96 | 97 | 1.0% |
| NGS -FANCM | 1 | 96 | 97 | 1.0% |
| NGS -FAT1 | 1 | 96 | 97 | 1.0% |
| NGS -ATRX | 1 | 130 | 131 | 0.8% |
| NGS -TRAF7 | 1 | 134 | 135 | 0.7% |
| NGS -NF1 | 1 | 138 | 139 | 0.7% |
| NGS -RUNX1 | 1 | 147 | 148 | 0.7% |
| NGS -PTCH1 | 1 | 149 | 150 | 0.7% |
| NGS -NFE2L2 | 1 | 154 | 155 | 0.6% |
| Met Exon 14 skip | 1 | 154 | 155 | 0.6% |
| Transcriptome-RET | 1 | 154 | 155 | 0.6% |
| NGS -KDM6A | 1 | 157 | 158 | 0.6% |
| NGS -DNMT3A | 1 | 162 | 163 | 0.6% |
| NGS -MAP2K4 | 1 | 166 | 167 | 0.6% |
| NGS -ROS1 | 1 | 170 | 171 | 0.6% |
| CNA-FGF3 | 1 | 171 | 172 | 0.6% |
| CNA-FGF19 | 1 | 172 | 173 | 0.6% |
| NGS -POT1 | 1 | 172 | 173 | 0.6% |
| NGS -MRE11 | 1 | 173 | 174 | 0.6% |
| NGS -MSI | 1 | 173 | 174 | 0.6% |
| CNA-CNBP | 1 | 174 | 175 | 0.6% |
| CNA-PDCD1 | 1 | 174 | 175 | 0.6% |
| CNA-PIM1 | 1 | 174 | 175 | 0.6% |
| CNA-RPN1 | 1 | 174 | 175 | 0.6% |
| CNA-CCND1 | 1 | 175 | 176 | 0.6% |
| CNA-CDKN2A | 1 | 175 | 176 | 0.6% |
| CNA-FGFR1 | 1 | 175 | 176 | 0.6% |
| CNA-MET | 1 | 175 | 176 | 0.6% |
| NGS -BRCA2 | 1 | 175 | 176 | 0.6% |
| NGS -JAK1 | 1 | 176 | 177 | 0.6% |
| NGS -MAP3K1 | 1 | 176 | 177 | 0.6% |
| NGS -AKT1 | 1 | 177 | 178 | 0.6% |
| NGS -BLM | 1 | 177 | 178 | 0.6% |
| NGS -ERCC2 | 1 | 177 | 178 | 0.6% |
| NGS -FANCA | 1 | 177 | 178 | 0.6% |
| NGS -MSH2 | 1 | 177 | 178 | 0.6% |
| NGS -SPOP | 1 | 177 | 178 | 0.6% |
| NGS -ERBB3 | 1 | 178 | 179 | 0.6% |
| NGS -FANCD2 | 1 | 178 | 179 | 0.6% |
| NGS -KRAS | 1 | 178 | 179 | 0.6% |
| NGS -PBRM1 | 1 | 178 | 179 | 0.6% |
| NGS -FLCN | 1 | 179 | 180 | 0.6% |
| NGS -HIST1H3B | 1 | 179 | 180 | 0.6% |
| NGS -KEAP1 | 1 | 179 | 180 | 0.6% |
| NGS -MAP2K1 | 1 | 179 | 180 | 0.6% |
| NGS -MEN1 | 1 | 179 | 180 | 0.6% |
| NGS -PIM1 | 1 | 179 | 180 | 0.6% |
| NGS -SPEN | 1 | 179 | 180 | 0.6% |
| NGS -TSC1 | 1 | 179 | 180 | 0.6% |
| dMMR/MSI-H | 1 | 191 | 192 | 0.5% |

Supplementary Table 5 Distribution of PDL1 TPS score among ROS1+ NSCLC

| TPS score | N | % |
| --- | --- | --- |
| 100 | 16 | 8.4% |
| 98 | 1 | 0.5% |
| 95 | 7 | 3.7% |
| 90 | 24 | 12.6% |
| 85 | 3 | 1.6% |
| 80 | 10 | 5.2% |
| 75 | 3 | 1.6% |
| 70 | 10 | 5.2% |
| 65 | 1 | 0.5% |
| 60 | 12 | 6.3% |
| 50 | 17 | 8.9% |
| 40 | 3 | 1.6% |
| 30 | 6 | 3.1% |
| 25 | 3 | 1.6% |
| 20 | 6 | 3.1% |
| 10 | 9 | 4.7% |
| 5 | 10 | 5.2% |
| 3 | 1 | 0.5% |
| 2 | 2 | 1.0% |
| 1 | 13 | 6.8% |
| 0 | 34 | 17.8% |
|  | 191 |  |
| 0 | 34 | 17.8% |
| 1-49 | 53 | 27.7% |
| 50 | 104 | 53.9% |

Supplementary Table 6 Distribution of PDL1 TPS score among ROS1+ tumors

| PD-L1 TPS score | N |  |  |
| --- | --- | --- | --- |
| 0 | 38 | 0 | 38/204 = 18.6% |
| 1 | 15 | 1-49 | 60/204 = 29.4% |
| 2 | 4 | 50 | 123/204 = 60.3% |
| 3 | 2 |  |  |
| 5 | 10 |  |  |
| 7 | 1 |  |  |
| 10 | 9 |  |  |
| 15 | 1 |  |  |
| 20 | 6 |  |  |
| 25 | 3 |  |  |
| 30 | 6 |  |  |
| 40 | 3 |  |  |
| 50 | 18 |  |  |
| 60 | 12 |  |  |
| 65 | 1 |  |  |
| 70 | 10 |  |  |
| 75 | 3 |  |  |
| 80 | 10 |  |  |
| 85 | 3 |  |  |
| 90 | 24 |  |  |
| 95 | 8 |  |  |
| 98 | 1 |  |  |
| 100 | 16 |  |  |
| Total | 204 |  |  |
